# Arterial adaptations to training among first time marathoners

**DOI:** 10.1186/s12947-016-0063-6

**Published:** 2016-05-26

**Authors:** Nicole M. Hafner, Christopher J. Womack, Nicholas D. Luden, Mikel K. Todd

**Affiliations:** James Madison University, Harrisonburg, VA 22807 USA

**Keywords:** Arterial remodeling, Endurance exercise, Arterial wall thickness, Lumen diameter, Intima-media thickness

## Abstract

**Background:**

Exercise training favorably alters arterial anatomy in trained limbs, though the simultaneous effects on passively trained arteries are unclear. Thus, brachial (non-trained limb), popliteal (trained limb) and carotid total wall thickness (TWT), wall-to-lumen ratios (W:L), intima-media thickness (IMT) and lumen diameters (LD) were compared between experimental (*n* = 14) and control (*n* = 11) participants before and after the experimental participants participated in marathon training.

**Methods:**

Arterial dimensions were measured with B-mode ultrasonography. Initial and final testing of VO_2max_ and running speed at 3.5 mmol lactate were measured in the experimental group.

**Results:**

VO_2max_ was unchanged by training, but running speed at 3.5 mmol lactate increased by 5 % (*p* = .008). Time by group interactions were observed for the brachial and popliteal measures (*p* < 0.05), but not the carotid. No changes were observed in the control group. Prior to the intervention the experimental group had larger LD in the brachial (*p* = .002) and popliteal arteries (*p* = .007) than controls; no other pre-testing differences were found. Following training, TWT declined in the brachial (pre = .99 ± .16 mm; post = .84 ± .10 mm; *p* = .007) and popliteal (pre = .96 ± .09 mm; post = .86 ± .11 mm; *p* = .005) arteries, characterized by a 0.07 mm decrease in brachial IMT (*p* = .032) and a non-significant 0.03 mm reduction in popliteal IMT. LD increased in the brachial (pre = 3.38 ± .35 mm; post = 3.57 ± .41 mm; *p* = .015) and popliteal (pre = 4.73 ± .48 mm; post = 5.11 ± .72 mm; *p* = .002) arteries.

**Conclusions:**

These data suggest that exercise-induced alterations in arterial dimensions occur in trained and non-trained limbs, and that adaptations may be dose dependent.

## Background

The cardioprotective effects of exercise can be partially explained by arterial remodeling that favors improved compliance and increased blood flow [11], adaptations that are inversely associated with hypertension [5], atherosclerotic disease [14], myocardial infarction and stroke [21]. More specifically, endurance exercise training improves vascular endothelial function by decreasing total arterial wall thickness (TWT), wall-to-lumen diameter ratios (W:L), intima-media thickness (IMT) and increasing lumen diameter (LD) [4, 18, 21, 27, 28]. While the evidence clearly suggests that these adaptations are manifested in the trained limbs [4, 10, 24–26], it is not clear how arterial dimensions in the non-trained limbs (e.g., arms during cycling or run training) and carotid arteries are affected by training [15, 22, 23, 25].

Cross-sectional data indicate that endurance trained runners and triathletes have larger femoral artery LD and smaller femoral IMT and IMT-to-LD ratio when compared to sedentary control participants [4]. Others report that femoral LD is larger in runners and cyclists when compared to sedentary controls and participants with spinal cord injuries [25] and that these differences exist regardless of gender or age [22]. Experimental evidence further suggests that arterial TWT [8, 27], IMT [4, 25] and W:L [8, 27] decreases in trained limbs, while LD increases [4, 8, 25, 28]. Together these data demonstrate that conduit arteries of active limbs undergo marked exercise training adaptations.

When compared to data from trained limbs, regular exercise appears to have a limited effect on the arteries in the non-trained limbs as well as the carotid arteries. In general, cross-sectional and longitudinal research suggest that brachial artery TWT [8, 22, 23] and W:L [8, 23] are lower among trained participants (i.e. cyclists, runners, etc.), but unlike the adaptations often found in the trained limbs, IMT [28] and LD [4, 8, 25, 27] are not different. Data related to the carotid arteries is also mixed, with studies showing that carotid IMT is unchanged after relatively short training programs [28, 31], but favorably influenced by longer duration training [24, 26, 27] when the accumulated stimulus is greater.

Some researchers propose that arterial remodeling due to exercise may result from repeated stimulation of locally functioning mechanisms (e.g., nitric-oxide (NO) system) [30], making it plausible that variations in the amount of exercise stimulus (e.g., blood flow) between arteries in the trained limbs, untrained limbs and carotid arteries accounts for the differences in adaptations. During leg cycling exercise, for example, blood flow to the lower extremities has been shown to be higher than it is in the upper extremities [3]. Other evidence indicates that blood flow to the head during exercise is only fractionally increased when compared to the limbs [3, 11].

A notable weakness within the cited research is a scarcity of data from studies that simultaneously assess arterial TWT, W:L, IMT and LD in trained limbs, non-trained limbs and the carotid artery [25], thus limiting the ability to assess how exercise volume influences remodeling in the trained limbs as well as arteries that are peripheral to the training stimulus. Thus, the present study was designed to compare TWT, W:L, IMT and LD in trained (popliteal), non-trained (brachial) and common carotid arteries between recreationally active and control group participants and to further evaluate whether 12 weeks of progressive marathon training among the recreationally active participants leads to further arterial remodeling.

It was hypothesized that the recreationally active (experimental group) would have significantly lower TWT, W:L and IMT, and greater LD than the control group in the brachial and popliteal arteries, but not the carotid artery prior to 12 weeks of marathon training. It was also hypothesized that there would be a significant group by time interaction characterized by reductions in TWT, W:L and IMT, and an increase in LD in all three arteries in the training group with no change in the control group following the marathon training.

## Methods

Fourteen male (*n* = 5) and female (*n* = 9) students from a university taught marathon running class and 11 inactive students volunteered to participate in this study. The number of recreationally active participants included in the experimental group exceeded the minimum sample size needed to elucidate initial and final training differences in the arterial dimensions of the trained limb with a statistical power of .80, an estimated effect size of 1.0 SD units, a two-tailed alpha level of 0.05 and an intra-class correlation of 0.80 between repeated measures [13]. All methods for the study were approved by the university’s Institutional Review Board prior to commencement.

Eligibility requirements for the experimental group included the ability to complete a five-mile continuous run prior to beginning the training program as well as having not previously run a marathon. After enrolling in the class, participants followed a 16-week marathon-training program. Initial and final-test data were collected before and after a 12-week segment within the training program. The program included four days of training per week and was divided into two phases. The first phase consisted of a 13-week training period that gradually increased the overall training volume by ~140 % between week one and week 13. The peak running volume occurred in weeks 12 and 13 with a total of 36 miles run during each of those weeks. The longest distance run during the first phase of training was 18 miles, which was performed on two occasions. The second phase of the training program consisted of a taper (reduced training volume) for three weeks. Compared to week 13, the running volume gradually decreased until the total weekly volume for the week before the marathon was decreased by 80 %.

During the third week of training, participants in the experimental group participants reported to the Human Performance Laboratory to complete informed consent, medical history questionnaire and the International Physical Activity Questionnaire (IPAQ; www.ipaq.ki.se). Initial and final testing was scheduled to accommodate other activities associated with the marathon class. For example, final testing was performed in the 15^th^ week since participant availability for testing during the 16^th^ week was limited due to traveling to the marathon. A familiarization trial for vascular ultrasonography was also performed during this visit. The experimental group reported to the laboratory later in the same week for ultrasonography of the brachial, popliteal and carotid arteries, as well as VO_2max_ and lactate threshold testing. After the 15^th^ week of the training intervention, the experimental group participants returned to the laboratory for IPAQ, VO_2max_, lactate threshold and ultrasonography measurements.

**Fig. 1:**
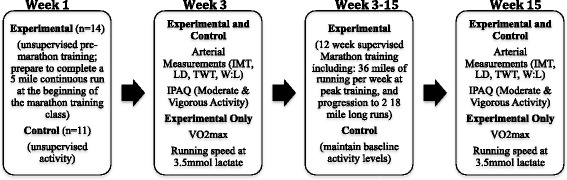
Marathon Training and Control Group Interventions and Data Collection Schematic. Schematic of research interventions and data collection

Control participants were recruited through a bulk e-mail detailing the research study and the participation requirements. During an initial visit to the Human Performance Laboratory, participants completed the informed consent, medical history questionnaire, and IPAQ. Ultrasound measurements of the carotid, brachial and popliteal arteries were performed during a second visit, which was completed by the end of week three of the study. A third visit was completed during weeks 15 and 16 of the study and included follow-up IPAQ and ultrasonography. Control group participants were also asked to maintain their physical activity throughout the duration of the study (Fig. 1). Moderate (breathe somewhat harder than normal) and vigorous (breathe much harder than normal) activity was defined in accordance with the criteria used on the IPAQ.

### Ultrasonography and arterial dimension measurements

For the ultrasound assessment, participants entered a quiet room with limited lighting and reclined in a supine position for 10 min. Following the rest period, right side carotid (1–3 cm inferior to the carotid bifurcation), brachial (1–3 cm superior to the anticubital fossa), and popliteal (1–3 cm superior to the popliteal fossa) images were obtained with high resolution, B–mode ultrasonography (Mindray, DC-6, 21 Mahwah, NJ) using a probe that was set at 10 MHz.

Analyses of the arterial images were performed with methods described by Veller et al. [34] and others [12, 34]. The only exception to the methodology described by Veller et al. [34] was that ImageJ software system (National Institute of Health; http://rsb.info.nih.gov/ij/) was used in place of the CalComp digitizing system. All ultrasound measurements preceded exercise testing and were recorded in mm. Arterial IMT was defined as the distance from the edge of the lumen-intima interface to the edge of the media-adventitia interface; and, TWT was defined as the distance between the deep lining of the intima (endothelium) and the superficial wall of the adventitia [34]. Vessel LD was defined as the distance from one edge of the lumen-intima interface to the edge of the opposing lumen-intima interface; and, W: L was calculated by dividing TWT by LD [34].

### VO_2max_ and lactate measurements

A graded exercise test was conducted to determine maximal oxygen uptake (VO_2max_), submaximal blood lactate concentrations, and lactate threshold in the experimental group. The treadmill protocol consisted of two discontinuous phases. Submaximal blood lactate concentrations and lactate threshold were assessed in the first phase. Once blood lactate levels exceed lactate threshold the treadmill was stopped and participants rested for a period of 15 min. The speed of running that immediately preceded lactate threshold was compared before and after the marathon training. Following 15 min of passive recovery, VO_2max_ was measured in the second phase of the exercise test.

### International physical activity questionnaire

The IPAQ was used to measure time (min per day) spent performing moderate activity and vigorous activity. The IPAQ was used to determine if physical activity was different between the groups and if within group activity was different between the beginning and the end of the study.

### Statistical analysis

Prior to hypothesis testing, all dependent variables were tested for normality using the Shapiro-Wilkes method. Variables that deviated significantly (*p* < 0.05) from the normal distribution (e.g., experimental pre-training carotid IMT) and the corresponding variables (e.g., control pre-training carotid IMT as well as control and experimental post-training carotid IMT) were normalized with log transformation (Log ^10). Two by two repeated measures ANOVA with one within subject factor (pre and post measurements) and one between subject factor (experimental and control groups) were used to identify significant interactions between the groups. When appropriate (time x group interaction; *p* < .05), a paired T-test was used to determine whether or not the dependent arterial dimensions changed over time within either group and an independent T-test was used to assess between group differences. T-test results were adjusted with the Bonferroni correction for multiple comparisons. A single researcher collected the ultrasound data. To assess researcher reliability, test-retest analysis (paired T-test; Pearson product moment correlation and coefficient of variation) was carried out on samples collected from an independent set of participants on two different occasions. Two-tailed alpha levels were used for all analyses and all data are presented as mean ± standard deviation.

## Results

Evaluation of the test-retest data collected from a separate group of participants indicated that, pre and post carotid measurements were significantly correlated (*r* = 0.98 to 1.00; *p* < .001), not significantly different (*p* > .05) and the coefficient of variation ranged from 0.20 to 2.26 %. Pre and post brachial measurements were significantly correlated (*p* < .001) not significantly different (*p* > .05) and the coefficient of variation ranged from 0.45 to 2.71 %. Pre and post popliteal measurements were significantly correlated (*p* < .001) not significantly different (*p* > .05) and the coefficient of variation ranged from 0.32 to1.89 %. Coefficients of variation are similar to those reported by Dinenno et al. [4].

There were no significant changes from initial to final measurements in either the experimental group or the control group in height or weight (Table 1). Given that there were no significant between group differences in weight and height during the study, changes in vessel dimensions were not likely attributable to changes in body size.

**Table 1:** Subject characteristics

	Marathon training group (*n* = 14)		Control (*n* = 11)	
	Initial	Final	Initial	Final
Age (yrs)	20.4 ± 1.6		19.4 ± 1.1	
Height (m)	1.72 ± 0.08	1.71 ± 0.08	1.63 ± 0.06	1.63 ± 0.05
Weight (kg)	65.48 ± 8.78	64.07 ± 7.49	64.92 ± 10.87	66.07 ± 10.94

### Physical activity and training

Initial testing combined moderate and vigorous activity values for the experimental and control groups were 361 ± 210 min/week and 31 ± 56 min/week (between group *p* = .000), respectively, and final testing values were 393 ± 136 min/week and 35 ± 69 min/week (between group *p* = .000). When the moderate and vigorous activity components were evaluated separately, the experimental group transitioned to spending significantly more time in vigorous exercise than moderate activity by the end of the study (pre moderate: 114 ± 123 vs. post moderate: 70 ± 83; *p* < 05; pre vigorous: 247 ± 102 vs. post vigorous: 323 ± 113; *p* = .038).

In the experimental group, VO_2max_ (ml/kg/min; L/min) did not differ between initial and final measurements. Running speed at lactate threshold increased significantly (*p* = .008) and lactate concentrations at a submaximal running speed decreased significantly (*p* = .001) (Table 2).

**Table 2:** Treadmill speed, lactate and VO_2_ in the experimental group

	Initial	Final
Lactate @ mean mph(7.54 ± 0.69)	2.54 ± .56^*^	1.87 ± .65
Treadmill speed (mph) @ 3.5 mmol lactate	7.75 ± .72^*^	8.15 ± .86
VO_2max_ (ml/kg/min)	43.32 ± 5.4	44.11 ± 5.0
VO_2max_ (L/min)	2.80 ± .56	2.82 ± .49

^*^ *p* < .01 within group initial and final training

### Arterial thicknesses, diameters and ratios: carotid artery

Initial testing carotid arterial measurements were not different between the groups. Likewise, initial- and final-carotid artery TWT, IMT, LD, and W:L were not different within either group (Table 3).

**Table 3:** Arterial wall thickness, diameter and ratio

	Marathon training group (*n* = 14)		Control (*n* = 11)	
	Initial	Final	Initial	Final
Carotid				
TWT (mm)	1.02 ± .07	.91 ± .16	.98 ± .06	.92 ± .06
IMT (mm)	.52 ± .08	.48 ± .10	.55 ± .05	.47 ± .09
LD (mm)	5.85 ± .86	5.90 ± .63	5.86 ± .97	5.74 ± .88
W:L	.18 ± .03	.16 ± .02	.17 ± .03	.16 ± .02
Brachial				
TWT (mm)	.99 ± .16^**^	.84 ± .10	.92 ± .14	.92 ± .01
IMT (mm)	.49 ± .09	.42 ± .08	.46 ± .10	.47 ± .07
LD (mm)	3.38 ± .35^*, **^	3.57 ± .41	2.98 ± .28	2.88 ± .29
W:L	.29 ± .04^**^	.24 ± .03	.31 ± .04	.32 ± .04
Popliteal				
TWT (mm)	.96 ± .09^**^	.86 ± .11	.89 ± .11	.88 ± .07
IMT (mm)	.47 ± .05	.44 ± .07	.46 ± .06	.48 ± .08
LD (mm)	4.73 ± .48^*, **^	5.11 ± .72	4.13 ± .51	4.15 ± .48
W:L	.20 ± .03^b^	.17 ± .03	.22 ± .02	.21 ± .02

*TWT* total wall thickness, *IMT* intima-media thickness, *LD* lumen diameter, *W: L* wall to lumen ratio

^*^ *p* < .025 between groups during initial training

^**^ *p* < .025 within group initial and final training

### Arterial thicknesses, diameters and ratios: brachial artery

Initial testing data shows that the experimental group had significantly larger brachial LD (*p* = .002) than the control group: no other initial-testing between group differences were found in terms of brachial TWT, IMT or W:L. There were no significant changes in the brachial artery measurements within the control group. Marathon training decreased brachial artery TWT (*p* = .007) and increased LD (*p* = .015). These combined changes resulted in a significant reduction in W:L (*p* = .000). Allowing for the Bonferroni statistical correction, IMT of the brachial artery tended to decrease in the experimental group (*p* = 0.032) (Table 3).

### Arterial thicknesses, diameters and ratios: popliteal artery (Figs. 2 and 3)

Initial testing data shows that the experimental group had significantly larger popliteal LD (*p* = .007) than the control group; no other initial-testing between group differences were found in terms of popliteal TWT, IMT or W:L. There were no significant changes in the popliteal artery measurements within the control group. Marathon training decreased popliteal TWT (*p* = .005) and increased LD (*p* = .007). There was also a significant reduction in W:L (*p* = .000). IMT of the popliteal artery did not change in the experimental group (*p* = .260) (Table 3).

**Fig. 2:**
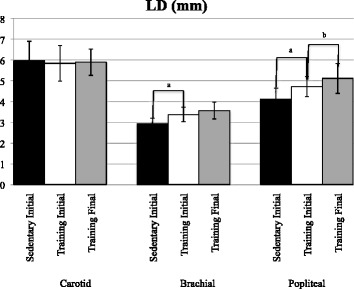
Lumen Diameter in Carotid, Brachial and Popliteal Arteries in Control and Experimental Participants. Data includes control subject dimensions before the intervention (Sedentary Initial), experimental participants before the intervention (Training Initial) and after marathon training (Training Final). Control post intervention results not shown (*p* > .05 compared to sedentary intial intervention data). Between group comparisons based on Bonferroni adjusted independent t-test results. Within group comparisons based on Bonferroni adjusted paired t-test results. ^a^ *p* < .025 between groups initial training. ^b^ *p* < .025 within group initial and final training

**Fig. 3:**
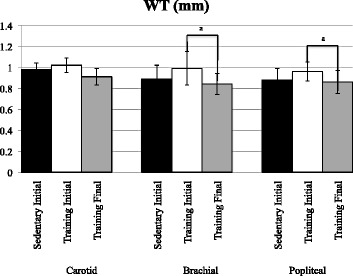
Arterial Wall Thickness in Carotid, Brachial and Popliteal Arteries in Control and Experimental Participants. Data includes control subject dimensions before the intervention (Sedentary Initial), experimental participants before the intervention (Training Initial) and after marathon training (Training Final). Control final intervention results not shown (*p* > .05 compared to sedentary initial intervention data). Between group comparisons based on Bonferroni adjusted independent t-test results. Within group comparisons based on paired Bonferroni adjusted t-test results. ^a^ *p* < .025 within group before and after training

## Discussion

### Physicial activity and training

This is the first study to simultaneously assess changes in TWT, W:L, IMT and LD in trained limbs (popliteal arteries), non-trained limbs (brachial arteries) and the carotid arteries after marathon training. As hypothesized, the experimental group experienced significant changes in TWT, W:L, and LD in the brachial artery and popliteal arteries after training and brachial IMT trended towards significance. IPAQ data showed that the experimental group was involved in significantly more moderate and vigorous activity than the control group before (*p* = .000) and after training (*p* = .000). This data also showed that vigorous activity increased significantly within the experimental group by the end of the training program (*p* = .038). Although VO_2max_ did not change in the experimental group, a 5 % increase in running speed at lactate threshold (*p* = .008) and lower lactate concentrations at a constant running speed (*p* = .001) was observed (Table 2). These data establish that the activity levels between the two groups were significantly different before and after training and that the marathon training elicited additional physiological adaptations.

### Initial-testing between group comparisons

The experimental group had significantly larger LD in the brachial (*p* = .002) and popliteal (*p* = .007) arteries than the control group during initial testing, whereas no differences were observed in the carotid LD. Initial testing TWT, W: L and IMT measurements were not different between the groups in any of the arteries (Table 3). The LD data is consistent with cross-sectional data presented by Rowley et al. [23] who found that LD in the brachial and femoral arteries of athletes was greater than inactive age-matched controls, but that carotid LD was not different. In contrast to the present study, these researchers [23] also reported that arterial TWT was lower in athletes when compared to controls. There are several possible explanations for this discrepancy.

Rowley et al. [23] proposed that exercise induced alterations in arterial dimensions are influenced by different mechanisms, with increases in LD caused by local factors (i.e. shear induced NO production) and reductions in TWT mediated by systemic mechanisms (e.g., arterial pressure). Similarly, Thijssen et al. [29, 32] found that in the weeks following spinal cord injury induced paralysis that the associated reduction in femoral LD stabilized after three weeks while TWT had not changed at three weeks but was significantly increased after 24 weeks. The authors concluded that changes in LD and TWT following spinal cord injury were on different time courses and that they may be influenced by different mechanisms [32]. Given the potential variability of the physical activity within the experimental group prior to initiating the marathon training program as well as the data suggesting that arterial adaptations may be dependent on the time course of the stimulation, it is not surprising that LD was found to be different between the two groups while TWT was not. Other evidence shows that TWT increases with age [18, 33] and that the increases in TWT are proportionally greater than age-related changes in LD [33]. These data further suggest that younger individuals with thinner vessel walls may experience a proportionally greater increase in LD compared to reductions TWT with training.

The between group differences found in the IPAQ and arterial dimension data provides evidence of a comparatively mild effect of physical activity (i.e., greater brachial and popliteal LD in the experimental group) that existed prior to beginning participation in the training intervention. In combination with the training effect seen in the experimental group, as discussed below, the initial between group differences suggest that arterial adaptations may be dose-dependent.

### Adaptations to training

Consistent with previous research we observed no training adaptations in the carotid artery [22–25] but found significant remodeling in the popliteal artery (trained limb) of the experimental group [4, 8, 23–25, 27] (Table 3). Popliteal adaptations included reductions in TWT (-10.4 %) and W: L (-15.0 %) as well as an increase in LD (8.0 %). No change was found in the popliteal IMT in the experimental group.

Similar to Green et al. [8], but in contrast to others [4, 8, 25, 27, 28], we found that brachial artery, or non-trained limb, dimensions were significantly altered by the 12 weeks of training (Table 3). Reductions were seen in TWT (-15.2 %) and W: L (-17.2 %), while LD increased (5.6 %) and IMT trended toward a significant reduction after allowing for the Bonferroni statistical correction (-14.3 %; *p* = 0.032). These results indicate that arterial adaptations to exercise training can be manifested in the non-trained limb (e.g. brachial artery) as well as the trained limb (e.g. popliteal artery). The data further suggest that adaptations may be dependent on training volume. This interpretation is supported by the evidence that the experimental group had greater LD in the brachial and popliteal arteries than the control group at the beginning of the study combined with the fact that LD further increased when vigorous training volume was increased. Our observations of increased LD in the brachial and popliteal arteries of the experimental group are consistent with data reported elsewhere [8]. Green et al. [8], for example, found that brachial LD increased by 4 % and popliteal LD increased by 12 %. Thijssen et al. [28] found that femoral LD increased by 7 % with training. The reduction in TWT and W: L in the brachial and popliteal arteries observed in this study are also consistent with Green et al. [8] who reported reductions in brachial TWT (14 %) and W: L (17 %) as well as reductions in popliteal TWT (10 %) and W: L (14 %).

Several mechanisms potentially contribute to arterial changes with training [27]. Included among these are, the endothelial derived NO-dilator system, exercise induced variations in systemic blood pressure as well as adaptations to oxidative stress and localized inflammation.

In the context of the present study, the role of NO is of particular interest given its responsiveness to variations in blood flow. Specifically, as blood flow increases, there is an increase in the shear stress acting on the vessel. With the increases in arterial blood flow that accompanies exercise, shear stress stimulates activity of the NO-dilator system, including the up-regulation of endothelial-derived NO synthase, to buffer the increased shear [16]. Although the precise mechanisms have yet to be elucidated, the increased activity of the NO-dilator system is associated with the modulation of platelet, macrophage and endothelial derived growth factors that favors increased LD and reductions in arterial TWT. This arterial remodeling continues until the shear stress is normalized and the NO activity returns back to baseline levels [16].

The simultaneous assessment of the carotid, brachial and popliteal arteries as performed in this study combined with blood flow and endothelial function data from other research provides a basis for explaining the potential role of shear stress and the NO-dilator system in arterial remodeling. There is clear evidence that blood flow to different regions of the body is varied during exercise [1, 2] and that it is further influenced by exercise mode [3] and intensity [7, 26]. While no data was found regarding blood flow to the legs and arms during running, Calbet et al. [3] showed that leg-only cross-country skiing lead to a second to eight-fold increase in femoral blood flow and a two to three-fold increase in subclavian blood flow. Other studies show that when the arms are immobilized during leg exercise, blood flow in the brachial artery increases as much as four-fold [7, 26] and that the higher flow is adequate to stimulate the NO-dilator system [9, 17]. In contrast, blood flow to the carotid artery during exercise increases to a lesser degree. Hellstrom et al. [10] found that the maximum change in blood flow to the head during exercise is 30-40 % higher than resting values. Tanaka et al. [26] found that blood flow to the active limbs during exercise increases in proportion to exercise intensity, while Goto et al. [7] showed that blood flow within a range of intensities is needed to stimulate the NO-dilator system and potentially alter arterial dimensions. These researchers reported that moderate intensity exercise (50 % Vo_2max_) is within the range required to enhance endothelial function in the non-training limb [7]. On the other hand, they found that low intensity exercise (25 % Vo_2max_) failed to stimulate the NO-dilator system, and any stimulatory effect associated with exercise at higher intensities (75 + % Vo_2max_) may be countered by an inflammatory response [7].

Given that leg exercise substantially increases blood flow in the legs and arms [3, 9], and that flow to the arms is adequate to invoke endothelial-derived dilation [9], it is plausible that the brachial and popliteal adaptations in LD, TWT, W: L found in the experimental group were mediated by the NO-dilator system. Although some researchers [4, 28] report that leg exercise is not associated with alterations in brachial artery dimensions, we propose that the adaptations found in the present study were a function of repeated bouts of moderate to high intensity exercise that were performed during 12 weeks of training. This exercise volume and intensity may have exceeded that used in studies that failed to show brachial artery adaptations to lower limb exercise training [4, 28]. Moreover, the fact that LD, but not TWT, was different between the two groups prior to training and that LD, TWT and W: L changed in the experimental group supports this position.

Despite the significant body of evidence indicating the NO-dilator system’s role in regulating arterial anatomy, the cyclical change in blood pressure that accompanies exercise training has not been ruled out as a mechanism responsible for these changes. Chronic elevations in transluminal pressure against the arterial wall, as seen in hypertension, activates pro-atherogenic endothelial cell phenotypes known to contribute to arterial wall thickening [6]. Exercise training, however, is associated with transient and cyclical increases in pressure; and, it has been proposed that the regulation of pro-atherogenic and anti-atherogenic genes is favorably altered by these cyclical variations in pressure [20]. Testing this theory is confounded by the fact that the exercise stimulus leading to increases in pressure are also associated with increases in blood flow and shear stress [20]. In one study, however, Rowley et al. [22] suggested that differences in brachial and femoral TWT between elite squash players and sedentary participants combined with a lack of any difference between arterial TWT in the dominant and non-dominant arms of squash players supports the systemic mechanism theory. Data from the study by Rowley et al. [22] support the need for more research designed to better explain the role that blood pressure plays in arterial remodeling.

An intriguing finding in the present study was that following the marathon training brachial IMT in the experimental group, when statistically adjusted for multiple comparisons, trended toward significance (*p* = 0.032) while popliteal IMT remained unchanged (*p* = 0.260). This result is in contrast with data presented by others [4, 8, 25] showing that IMT decreased in the trained limb as a result of exercise training. It is possible that the discrepancy is due to localized inflammation in the lower extremities associated with a relatively high volume of training (i.e., 36 total miles, inclusive of an 18 mile run) that occurred during the week prior to data collection. In response to this training load, the participants may have experienced sufficient micro-trauma in the legs to stimulate localized inflammation. This explanation is supported by limited data [7, 19] and constitutes an area of research that has been identified as one in need of further investigation [30].

An alternate explanation for the lack of change in popliteal IMT may be related to variations in oscillatory pattern of blood flow in limbs during exercise. Thijssen et al. [31] and Tinken et al. [32] demonstrated that the NO-dilator activity is directly related to ratio of antegrade to retrograde flow; and vessel occlusion reduces the ratio in favor of retrograde flow and diminished NO-dilator activity. The reduction in NO-dilator activity is proportional to the degree of occlusion, and even a mild occlusion (i.e., cuff pressure at 25 mmHg) is adequate to stimulate a significant change in the ratio and NO-dilator activity. Based on this data, it seems plausible that during the loading phase of the running stride, occlusion of the conduit arteries in the leg would favor a reduction in the antegrade to retrograde flow and perhaps attenuate NO-dilator activity and any related effect on IMT.

### Limitations

A limitation of the study is that the initial training before the marathon class began was unsupervised. Therefore it is not clear as to what type or amount of training was being done initially. Another limitation of the study is that due to the unique loading of the arms and legs during running, the results are not generalizable to cycling (static loading of the arms) or activities that place a greater load on the arms (cross country skiing, circuit training).

## Conclusion

Simultaneous comparison of TWT, W: L, IMT and LD in carotid, brachial and popliteal arteries show that LD in the brachial and popliteal arteries is different between young, recreationally active participants and sedentary controls prior to marathon training. These data also show that arterial dimensions in the trained (popliteal) and non-trained limb (brachial) are further altered with 12 weeks of marathon training but no differences are seen in the carotid artery. The combined evidence suggests that arterial dimension adaptations to training are dependent on training volume and that higher exercise intensities may extend the influence of localized mechanisms into arteries that are peripheral to the trained limbs. These data also indicate that further study is needed to explore the interactions between the antegrade to retrograde flow ratio and the NO-dilator system as well as the role that localized inflammation that occurs with intense training plays in arterial dimension adaptations.

## Abbreviations

IMT, intima-media thickness; IPAQ, international physical activity questionnaire; LD, lumen diameter; NO, nitric oxide; SD, standard deviation; TWT, total wall thickness; VO_2max_, maximal oxygen consumption; W: L, wall-to-lumen ratio.
